# Patterns of Bacterial Infections Following Orthopedic Surgeries: A Retrospective Analysis

**DOI:** 10.7759/cureus.112197

**Published:** 2026-07-07

**Authors:** Dana Avraham, Alexander Kaban, Amir Herman, Haim Itzhak, Nir Gafni, Maria Oulianski

**Affiliations:** 1 Orthopedics, Kaplan Medical Center, Rehovot, ISR

**Keywords:** infection rates, orthopedics surgery, post operative complication, prophylactic antibiotics, surgical site injection

## Abstract

Background and objective

Surgical site infections (SSIs) following orthopedic procedures are associated with significant morbidity and continue to pose an ongoing clinical challenge. Pathogen patterns vary by surgical category and may not be adequately covered by standard perioperative antibiotic prophylaxis, which has remained largely unchanged for decades. This study aimed to characterize pathogen distribution patterns across different orthopedic surgical categories and to evaluate whether current prophylactic regimens align with the observed microbiological profile.

Methods

This retrospective single-center study included 98 patients who developed SSIs involving implanted hardware following elective, semi-elective, or trauma-related orthopedic surgeries. Inclusion criteria required a positive wound culture obtained using a sterile technique during the first surgical debridement.

Results

The mean age of the cohort was 68.5 years. The mean interval from primary surgery to debridement and irrigation was 67.1 days. Patients undergoing elective arthroplasty presented at a chronic stage, with a mean interval of 132 days. The most frequently isolated pathogen was methicillin-sensitive *Staphylococcus aureus* (MSSA; n = 23, 23.5%, 95% confidence interval (CI): 16.2%-32.9%). Gram-negative organisms predominated in spine surgeries, accounting for 69% of isolates (n = 9/13, 95% CI: 39.5%-88.1%). The trauma and femur fracture groups were predominantly infected with Gram-positive organisms. Mixed Gram-positive and Gram-negative bloodstream infections were detected in 14% of blood cultures (n = 14, 95% CI: 8.7%-22.6%), exclusively in the femur fracture and trauma groups.

Conclusions

Pathogen patterns differ substantially across orthopedic surgical categories. The predominance of Gram-negative organisms in spine SSIs suggests that further investigation of pathogen-specific prophylactic strategies in spine surgery may be warranted. Prospective studies are needed to determine whether targeted adjustments to prophylactic regimens can effectively decrease SSI rates in spine surgery.

## Introduction

Surgical site infections (SSIs) following orthopedic procedures are associated with prolonged hospitalization, increased healthcare costs, and adverse patient outcomes. A five-year study reported an SSI prevalence of 2.55% in orthopedic surgeries, with extended surgical duration and comorbidities identified as independent risk factors [1]. These infections are closely linked to the implantation of foreign materials - prostheses, screws, plates, and other hardware - which provide surfaces for bacterial adhesion and biofilm formation [2-4]. Biofilms consist of microbial cells embedded within an extracellular polymeric substance (EPS) matrix that adheres strongly to metal implant surfaces. This EPS matrix serves as a physical and chemical barrier, limiting antibiotic penetration and reducing disinfectant effectiveness, thereby contributing to persistent infections that are difficult to eradicate with standard treatment [2-4].

The most common pathogens associated with orthopedic SSIs are *Staphylococcus aureus* and coagulase-negative staphylococci, both of which are capable of developing antibiotic resistance [5,6]. Other reported causative organisms include *Staphylococcus epidermidis*, aerobic streptococci, and anaerobic cocci. Perioperative antibiotic prophylaxis, together with aseptic technique and preoperative skin decontamination, remains the cornerstone of SSI prevention; when used appropriately, antibiotic prophylaxis alone can reduce SSI rates by up to 80% [7-9]. In arthroplasty, studies have evaluated the efficacy of cephalosporins compared with glycopeptides, cephalosporins compared with penicillin derivatives, and first-generation versus second-generation cephalosporins [7,10].

The current standard regimen consists of 2 g of a first-generation cephalosporin administered 30 minutes before incision, followed by two additional 1 g doses. In arthroplasty, a perioperative dose of aminoglycosides is added to extend coverage against Gram-negative organisms [11,12]. This regimen has remained largely unchanged for decades, although its adequacy across different surgical categories continues to be examined [13-17]. The purpose of this study was to evaluate the distribution of bacterial pathogens responsible for hardware-associated SSIs across different orthopedic surgical categories and to descriptively compare these microbiological profiles with the expected spectrum of standard perioperative antibiotic prophylaxis.

## Materials and methods

This retrospective single-center study was performed at Kaplan Medical Center and reported in accordance with the STROBE (Strengthening the Reporting of Observational Studies in Epidemiology) guidelines. The study received approval from the Kaplan Medical Center Helsinki Committee Ethics Committee (approval no. KMC-0013-22) and was conducted in compliance with the Declaration of Helsinki. The requirement for informed consent was waived due to the retrospective nature of the study design.

**Figure 1:**
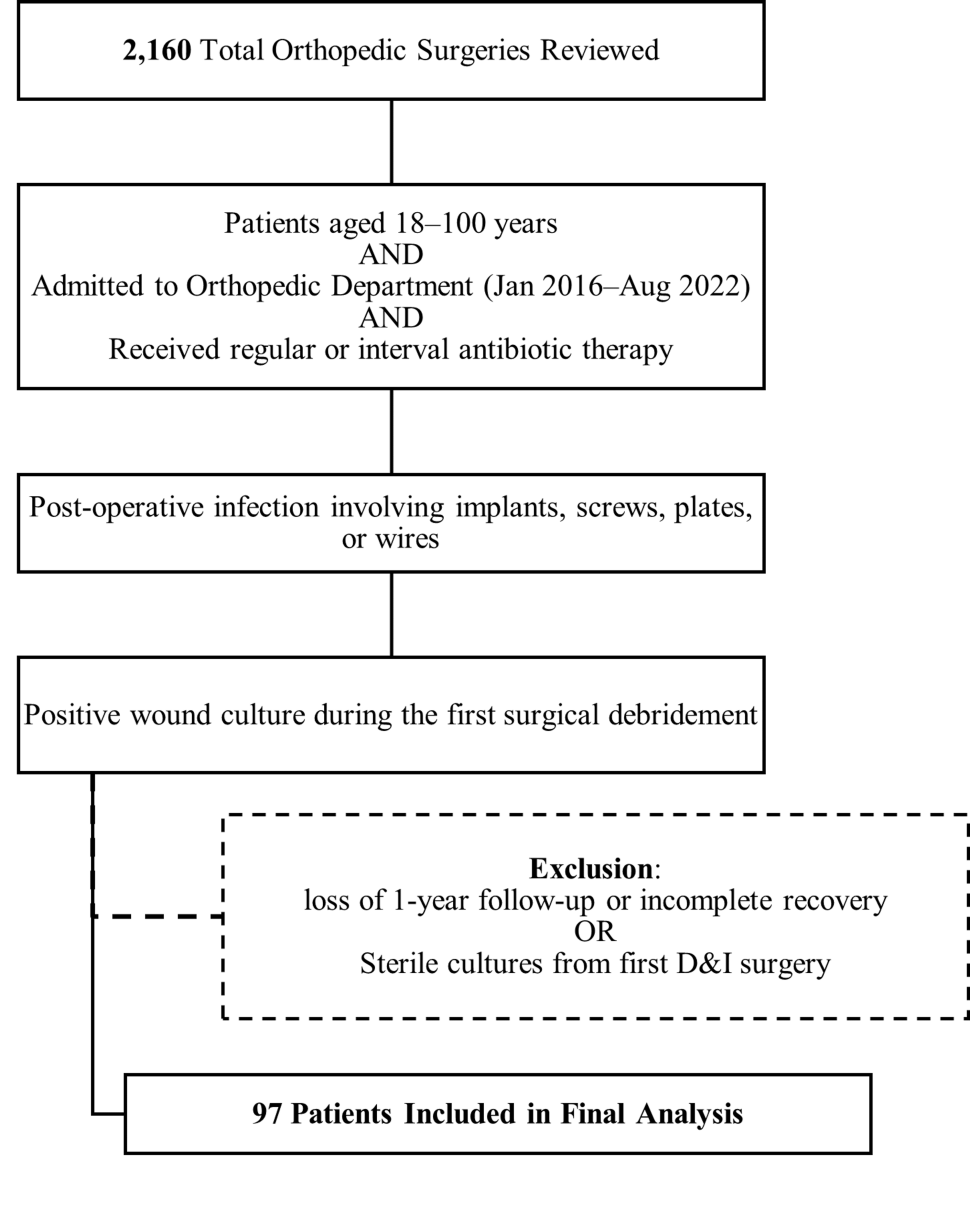
Flowchart of patient inclusion and exclusion criteria D&I: debridement and Irrigation

A total of 2,160 patients underwent screening, of whom 98 fulfilled the eligibility criteria and were included in the final analysis. The cohort comprised male and female patients aged 18-100 years who were admitted to the orthopedic department between January 2016 and August 2022. The complete inclusion and exclusion criteria applied to this cohort are presented in Table 1. Figure 1 shows a flowchart depicting patient inclusion and exclusion criteria.

**Table 1:** Inclusion and exclusion criteria ^*^SSI was clinically defined according to the CDC/NHSN criteria for deep incisional or organ/space SSI, requiring purulent drainage, localized pain/tenderness, or imaging evidence of infection in the presence of orthopedic hardware. ^**^Consequently, culture-negative deep tissue cases were excluded from this cohort, though cases with negative blood cultures but positive tissue cultures were retained SSI: surgical site infection; CDC/NHSN: Centers for Disease Control and Prevention/National Healthcare Safety Network; ACL: anterior cruciate ligament

Inclusion criteria	Exclusion criteria
Admitted to the orthopedic department between January 2016 and August 2022	Lacked complete one-year follow-up
Aged between 18 and 100 years (males and females)	Recovered before the one-year follow-up was reached
Underwent elective, semi-elective, or trauma-related orthopedic surgery	Infection was found initially in revision surgery
Developed a postoperative infection involving implanted hardware (e.g., implants, screws, plates, non-absorbable wires; see Figure 1)^*^	Underwent tendon reconstruction as the primary surgery (e.g., ACL reconstruction)
Received antibiotic therapy	
Had a positive wound culture obtained by sterile technique^**^	

The primary outcome was the distribution of Gram-positive and Gram-negative pathogens across surgical categories. These categories were operationally defined as follows: (1) trauma: acute internal/external fixation of non-spine fractures; (2) spine: elective or emergent spinal instrumentation/decompressions; (3) femur fractures: proximal or shaft femur fixation in elderly or polytrauma patients; and (4) elective arthroplasty: primary total hip or knee replacements for osteoarthritis (OA). Secondary outcomes included the time interval from primary surgery to debridement and irrigation surgery, the interval from infection identification to reoperation, and the distribution of pathogens in blood versus tissue cultures.

Only cultures obtained during the first surgical debridement were analyzed, as these were considered the most reliable representation of the infecting organism. Cultures from subsequent debridements, bedside swabs, and outpatient clinic swabs were excluded. Wound cultures were collected using sterile techniques during the first debridement, specifically from deep tissue or joint fluid samples obtained intraoperatively. Blood cultures were drawn preoperatively where clinically indicated, following standard aseptic protocols. All samples were transported to the microbiology laboratory within one hour of collection. For microbiological analysis, separate denominators were calculated for tissue samples and blood cultures based on the number of patients who underwent each test. For the handling of polymicrobial cultures, if a single sample yielded multiple distinct organisms, each isolated pathogen was recorded and counted individually toward the total pathogen distribution to capture the full microbiological burden.

Data were extracted from medical records and included patient demographics, medical history, surgical details (elective, semi-elective, trauma, open fractures), and preoperative risk factors including comorbidities, immune status, and use of perioperative antibiotic prophylaxis. All included patients received perioperative antibiotic prophylaxis in accordance with the standard institutional prophylactic regimen described in the introduction. The clinical course was documented for complications, repeat surgeries, and treatment durations.

Data analysis was performed using SPSS Statistics for Windows (version 25.0; IBM Inc., Armonk, NY). Descriptive statistics were calculated for all variables: means and standard deviations (SD) for continuous variables (age, time intervals) and frequencies and percentages for categorical variables (pathogen types, surgical procedures, comorbidities). The distribution of Gram-positive and Gram-negative bacteria was analyzed across surgical groups using cross-tabulation and percentage calculations. To account for sample size variations and provide an estimate of precision, 95% confidence intervals (CI) for proportions were calculated using the Wilson score method. Temporal trends in bacterial patterns were examined by year across the study period. Time-to-event variables, including the interval from primary surgery to debridement and irrigation, were calculated and presented as means. No formal inferential statistical tests were performed; all comparisons are descriptive.

## Results

A total of 98 cases were included in the study, while 15 were excluded because the procedures - revision surgeries and ACL reconstructions - were outside the scope of the disciplines examined. The mean age of participants was 68.5 years, with the trauma group being the youngest (mean age: 56.0 years) and the femur fracture group being the oldest (mean age: 79.6 years). On average, patients were readmitted to the orthopedic department 62.2 days following the primary surgery. The mean interval between the primary surgery and debridement and irrigation surgery was 67.1 days. The average duration from initial identification of infection to reoperation was 4.9 days.

The most frequently isolated pathogen was methicillin-sensitive* Staphylococcus aureus *(MSSA)*, *accounting for approximately 23% of cases (23%, 95% CI: 16.2%-32.9%; n = 23), followed by *Enterobacter cloacae *(10%, 95% CI: 5.6%-17.8%; n = 10), *Pseudomonas aeruginosa *(10%, 95% CI: 5.6%-17.8%; n = 10), *Escherichia coli *(9%, 95% CI: 4.9%-16.5%; n = 9), and *Staphylococcus epidermidis *(9%, 95% CI: 4.9%-16.5%; n = 9). Less commonly isolated pathogens (2-5% each) included* Enterococcus faecalis, Acinetobacter baumannii, Proteus mirabilis, *methicillin-resistant* Staphylococcus aureus *(MRSA)*, *and *Serratia marcescens*. Several other pathogens were identified in approximately 1% of cases.

In trauma patients, blood cultures revealed Gram-positive pathogens in 20 cases (56%, 95% CI: 39.6%-70.5%), compared with 19 (53%, 95% CI: 37.0%-68.0%) from tissue samples, while five (14%, 95% CI: 6.1%-28.9%) showed no microbial growth. In the spine group, four isolates (31%, 95% CI: 11.9%-60.5%) were Gram-positive, and nine (69%, 95% CI: 39.5%-88.1%) were Gram-negative, with a similar descriptive trend toward Gram-negative isolates observed in tissue samples (61.5%, 95% CI: 33.3%-83.3%; n = 8 Gram-negative vs. 38.5%, 95% CI: 16.7%-66.7%; n = 5 Gram-positive). Given the small size of this subgroup (n = 13), these proportions represent a localized observation rather than a generalizable pattern. For the femur fractures and the OA knee and femur replacement group, the percentage of Gram-positive pathogens in blood samples was comparable.

However, in tissue samples, the OA knee and femur replacement group showed a higher proportion of Gram-negative pathogens (50%, 95% CI: 28.0%-72.0%; n = 8) than Gram-positive pathogens (31%, 95% CI: 14.2%-55.6%; n = 5). In contrast, the femur group showed the opposite pattern in tissue samples, with Gram-positive pathogens predominating (52%, 95% CI: 35.2%-67.5%; n = 17) over Gram-negative pathogens (4%, 95% CI: 27.2%-59.2%; n = 14). No concurrent Gram-positive and Gram-negative infections were observed in tissue samples across all surgery types. In contrast, 14% (95% CI: 8.7%-22.6%; n = 14) of blood samples revealed mixed Gram-positive and Gram-negative bloodstream infections, which were detected exclusively in the trauma and femur fracture groups. Table 2 summarizes the distribution of pathogens across the different groups, highlighting the proportions of Gram-positive and Gram-negative organisms.

**Table 2:** Gram-type of bacteria according to surgery type Numbers in parentheses (n = X) represent the actual number of patients in each category OA: osteoarthritis; CI: confidence interval

Variable		All (n = 98)	Trauma (n = 36)	Spine (n = 13), % (95% CI)	Femur (n = 33), % (95% CI)	OA knee/hip (n = 16), % (95% CI)
	Number of patients	98	36	13	33	16
	Age	68.5	56	66.9	79.6	71.8
Gram from blood, % (95% CI)	+	46% (36.4-55.8) (n = 45)	56% (39.6–70.5) (n = 20)	31% (11.9–60.5) (n = 4)	42% (27.2–59.2) (n = 14)	44% (23.1–67.4) (n = 7)
	-	40% (30.7–49.7) (n = 39)	33% (20.0–50.0) (n = 12)	69% (39.5–88.1) (n = 9)	27% (14.8–44.2) (n = 9)	56% (32.6–76.9) (n = 9)
	Both	14% (8.7–22.6) (n = 14)	11% (4.4–25.3) (n = 4)	0% (0.0–22.1) (n = 0)	30% (17.3–47.1) (n = 10)	0% (0.0–18.5) (n = 0)
Tissue during surgery, % (95% CI)	+	47% (37.3–56.7) (n = 46)	53% (37.0–68.0) (n = 19)	38% (16.7–66.7) (n = 5)	52% (35.2–67.5) (n = 17)	31% (14.2–55.6) (n = 5)
	-	43% (33.5–52.7) (n = 42)	33% (20.0–50.0) (n = 12)	62% (33.3–83.3) (n = 8)	42% (27.2–59.2) (n = 14)	50% (28.0–72.0) (n = 8)
	None	10% (5.6–17.8) (n = 10)	14% (6.1–28.9) (n = 5)	0% (0.0–22.1) (n = 0)	6% (1.7–19.6) (n = 2)	19% (6.8–42.9) (n = 3)

## Discussion

The predominance of MSSA as the most frequently isolated pathogen (n = 23, 23.5%, 95% CI: 16.2%-32.9%) aligns with existing literature on SSIs in orthopedic settings [18], consistent with the Gram-positive focus of standard first-generation cephalosporin prophylaxis. The central finding of this study, however, was the distribution of Gram-negative pathogens in spine surgeries, where Gram-negative organisms accounted for 69% (n = 9/13) of isolates. Although CIs were calculated, the small size of the spine subgroup (n = 13) resulted in wide intervals (95% CI: 39.5%-88.1%), indicating that this finding represents an imprecise, descriptive trend rather than a generalizable predominance. While it questions the coverage of standard first-generation cephalosporins across all subgroups, this observation serves strictly as a hypothesis-generating finding rather than definitive clinical evidence.

This observation raises a direct question about prophylactic coverage for spine cases. First-generation cephalosporins provide limited activity against Gram-negative pathogens; if these organisms predominate in spine SSIs, the standard regimen may not provide adequate coverage for this group. Shen et al. [19] described similar challenges in Gram-negative SSIs following tibial plateau fracture fixation. Fletcher et al. [20], in contrast, found no evidence supporting Gram-negative antibiotic coverage perioperatively or postoperatively except in open fractures - a point worth noting given that the spine cases in this study were not open fractures.

Elective arthroplasty patients were unique in presenting at a chronic stage, with a mean of 132 days to debridement and irrigation surgery. Trauma and femur fracture groups presented in the subacute stage. Costerton [21] described the role of biofilm in device-related infections: biofilms form a protective barrier that renders pathogens resistant to antibiotics and standard antimicrobial treatment, making infections more persistent. One possible explanation for the delayed presentation observed in arthroplasty patients is biofilm formation on prosthetic surfaces. Biofilm-associated organisms may remain clinically silent for prolonged periods before infection becomes apparent. Differences in implant surface area, host immune response, and surgical technique may also contribute.

Femur fracture patients showed the highest rate of mixed Gram-positive and Gram-negative bacteremia (30%, n = 10), with trauma patients also showing mixed infections (11%, n = 4); no mixed infections were detected in spine or arthroplasty groups. No concurrent infections were observed in tissue samples across any group. This discrepancy may reflect differences in the pathophysiology of systemic versus localized infection, or it may reflect limitations of culture sensitivity - tissue cultures may miss organisms that are detectable in blood. Tan et al. [16] showed that prophylactic strategies tailored to patient-specific risk factors, including comorbidities and local susceptibility patterns, can reduce infection rates in arthroplasty; a similar individualized approach may be warranted in trauma and femur fracture patients with mixed bacteremia.

In arthroplasty, the combination of first-generation cephalosporins and aminoglycosides provides coverage against both Gram-positive and Gram-negative pathogens. The relatively low Gram-negative rates in this group are consistent with the use of that dual regimen. Argaw et al. [15] and Bandalović et al. [17] both demonstrated the value of broad-spectrum prophylaxis in reducing Gram-negative SSIs in orthopedic procedures. Whether a similar dual approach could benefit spine patients, who showed the opposite pathogen pattern, is a question this study cannot answer but one that warrants prospective investigation.

First-generation cephalosporins remain the standard perioperative regimen across most orthopedic categories. The data from this study do not provide grounds for a universal change, but they do identify a specific subgroup, spine surgery, where the pathogen burden is predominantly Gram-negative and may warrant further investigation. Argaw et al. [15] noted that routine prophylaxis protocols may require re-evaluation in settings where emerging Gram-negative pathogens are prevalent; the spine group in this cohort is consistent with that concern.

This study has notable strengths, including its clinically relevant research question, use of deep intraoperative cultures from the first debridement, and description of microbiological patterns across surgical categories. However, it has several limitations. First, this study is strictly descriptive and evaluates only patients who developed SSIs, meaning it lacks a non-infected denominator. Consequently, we cannot calculate absolute infection rates, evaluate the protective efficacy of current prophylactic regimens, or assess direct prophylaxis exposure and susceptibility data. Second, the retrospective, single-center design limits generalizability; findings reflect the pathogen ecology of one institution and may not apply to other centers. Third, only cultures from the first surgical debridement were analyzed; cultures from subsequent debridements, bedside swabs, or outpatient samples were excluded, potentially underrepresenting infections with atypical presentations or delayed microbial growth. Also, discrepancies between blood and tissue cultures introduce methodological uncertainty about the true pathogen burden in each group. Despite these limitations, the findings provide a valuable hypothesis-generating framework for targeted prospective investigation.

Prospective studies with larger cohorts, advanced molecular diagnostics, and formal statistical analyses of confounders are needed to clarify whether the predominance of Gram-negative organisms in spine SSIs warrants modification of prophylactic regimens. Strategies targeting biofilm formation on surgical implants - including antimicrobial coatings and local antibiotic delivery - may offer additional avenues for reducing SSI rates across all surgical categories.

## Conclusions

This study highlights the distinct pathogen distribution patterns observed among patients with established SSIs across different orthopedic categories. However, because this retrospective descriptive study lacks non-infected denominators, prophylaxis exposure data, antimicrobial susceptibility data, and formal outcome comparisons, it cannot assess the effectiveness or adequacy of current protocols. Consequently, the observed trend toward Gram-negative organisms in spine cases is strictly hypothesis-generating and should not be interpreted as definitive evidence. Future large-scale prospective studies are required to validate these findings and determine whether modifications to current prophylactic strategies are warranted.
